# The Prevalence of Vitamin D Deficiency and Associated Factors Among Subfertile Women: An Initial Experience From Northern Sri Lanka

**DOI:** 10.7759/cureus.72094

**Published:** 2024-10-22

**Authors:** Raguraman Sivalingarajah, Vithegi Kesavan, Pethirupillai A Coonghe, Sheron A Vethanayagam, Sivalingam Jeyaluxmy, Kaviranjan Dhushyanthy, Shathana Paramanathan

**Affiliations:** 1 Obstetrics and Gynaecology, University of Jaffna, Jaffna, LKA; 2 Chemical Pathology, Jaffna Teaching Hospital, Jaffna, LKA; 3 Community and Family Medicine, University of Jaffna, Jaffna, LKA; 4 Center for Digital Epidemiology, University of Jaffna, Jaffna, LKA; 5 Surgery, University of Jaffna, Jaffna, LKA

**Keywords:** prevalence, sri lanka, sub-fertile women, vitamin d, vitamin-d deficiency

## Abstract

Introduction: Subfertility is a global burden. Low vitamin D status has been associated with an increased risk for subfertility. Even though the prevalence of vitamin D deficiency is very high among women in Sri Lanka, not much importance is given to addressing the issue, especially among subfertile women. Moreover, no studies have been reported on the prevalence of vitamin D in subfertile women in Sri Lanka. Thus, the current study aimed to assess the prevalence and associated risk factors among subfertile women in the northern part of Sri Lanka.

Methodology: This was a cross-sectional study comprising an interviewer-administered questionnaire and a laboratory analysis of vitamin D conducted on 371 subfertile women above 18 years of age who attended the subfertility clinic at the Professorial Unit in the Jaffna Teaching Hospital, Jaffna, Sri Lanka, between January 2023 to October 2023. Considering the time limitation and sample size, subfertile women attending during the study period were included without any sampling technique. Competitive chemiluminescence immunoassay for 25 hydroxy vitamin D (25(OH)D) was used to estimate the vitamin D level, and an external quality assessment (EQA) standard was used to categorize the level into deficient, insufficient, and sufficient. Chi-squared and ANOVA tests were used to assess the factors associated with vitamin D levels using IBM SPSS Statistics for Windows, Version 21.0 (Released 2012; IBM Corp., Armonk, New York, United States).

Results: The mean age of subfertile women was 33.7 years (SD=6.7), and nearly half of them (n=173, 46.6%) were in the age group of 30-40 years. All the participants (n=371, 100%) were Sri Lankan Tamil, the majority (n=289, 77.9%) of them were Hindus, and nearly three-quarters of the participants (n=269, 72.5%) were unemployed. Moreover, almost 90% (n=334) of the subfertile women never estimated their vitamin D level prior to the study. The study revealed that only 36.4% (n=135) of subfertile women had adequate vitamin D levels, whereas 63.4% (n=235) had inadequate and low BMI levels, and intake of fish and eggs was statistically associated with adequate vitamin D levels.

Conclusion: The study recommends a routine assessment of vitamin D levels, periodical monitoring of vitamin D levels for all subfertile women with particular attention towards the obese and overweight category, and a combination of pharmacological and dietary interventions to improve vitamin D levels, which might benefit the fertility outcome.

## Introduction

The World Health Organization defines subfertility as a global reproductive health problem that affects one in six couples [1]. Subfertility is a disease of the reproductive system in which a woman fails to achieve pregnancy after 12 months or more of regular unprotected sexual intercourse. Vitamin D deficiency is associated with numerous diseases, including diabetes, autoimmune diseases, cardiovascular diseases, and cancer [2]. However, despite inconclusive data, vitamin D has also been an important factor for subfertility; a recent systematic review demonstrated that vitamin D supplementation successfully improved the clinical pregnancy rate of subfertile women [3,4].

Vitamin D is a steroid hormone that plays a significant role in the reproductive physiology of both genders, and its metabolizing enzymes are present in reproductive tissues such as the ovaries, testes, uterus, placenta, and endocrine organs such as the pituitary gland, and hypothalamus [5]. Moreover, vitamin D stimulates the production of essential reproductive hormones such as estradiol and progesterone. Several studies conducted in animals and humans have suggested that vitamin D plays a role in follicular genesis, steroidogenesis, and endometrial regulation in the female gender [6]. It plays a crucial role in subfertility, and especially low vitamin D levels are found in subfertile women [7]. Polycystic ovary syndrome (PCOS) is one of the major causes of subfertility, especially in South Asian women, and vitamin D deficiency is frequently observed in PCOS women [8]. High levels of vitamin D are associated with endometriosis, and low levels are associated with subfertility [6].

Even though sunlight exposure is high in Asian countries, vitamin D deficiency is commonly observed in people living in this area due to other factors influencing the vitamin D level [9]. A high prevalence of vitamin D deficiency has been reported in countries that get high sunlight compared to countries that get low sunlight [10]. Vitamin D deficiency was high among Sri Lankan adult women; for instance, in a study conducted in Southern Sri Lanka (2013), vitamin D deficiency was observed among 56.2% of community-dwelling healthy women [11]. Even though it is very common and the literature suggests its negative impact on subfertility, it is a poorly addressed health issue in Sri Lanka [12]. Studying the distribution of vitamin D deficiency and socio-behavioral determinants among subfertile women is crucial to implementing effective strategies. Further, these findings will help to address the unmet health burden and provide a cost-effective solution in a country like Sri Lanka, where the majority refers to low-resourced health settings. Thus, this study aims to assess the prevalence and associated risk factors of vitamin D deficiency among subfertile women in the northern part of Sri Lanka.

## Materials and methods

This was an institutional-based cross-sectional descriptive study conducted among women above 18 years of age who attended the Subfertility Clinic at the Professorial Unit in the Jaffna Teaching Hospital, Jaffna, Sri Lanka, from January 2023 to October 2023. The Ethics Review Committee, Faculty of Medicine, University of Jaffna approved the study (approval number: J/ERC/23/145/NDR/0293). An information sheet was provided to each participant, and written informed consent was obtained before collecting the data.

Participant selection and sample size

Women who had already been diagnosed with vitamin D deficiency that may impact calcium or vitamin D metabolism and patients already on regular intake of vitamin D or calcium supplementation from three months or more prior to the study were excluded from this study. Barring these exclusion criteria, all women who were diagnosed as subfertile during the study period were included in this study.

Since no relevant studies were found related to the prevalence of vitamin D deficiency in Sri Lankan subfertile women, a prevalence of 68.4%, from a study conducted in Asia [13], was considered to calculate the sample size; the calculated sample size was 371.

Data collection

Interview-Administered Questionnaire

An interview-administered questionnaire (see Appendices) was used following bilingual translation and content validation to collect data, which consists of questions to assess the sociodemographic factors, subfertility factors, and factors associated with vitamin D deficiency.

Laboratory Analysis

A volume of 2 ml of blood was collected from the participants by trained nurses using the standard vacutainer technique. The serum was separated immediately after a firm clot was formed, and the separated serum was stored at -20 °C until analysis. The levels of 25 hydroxy vitamin D (25(OH)D), a form of Vitamin D commonly measured to assess vitamin D status, were calculated and used in the analysis. The total 25(OH)D was analyzed on Vitros 5600 (QuidelOrtho Corporation, San Diego, California, United States) by competitive chemiluminescence Immunoassay using dedicated reagents. Three levels of Internal Quality Controls from Bio-Rad were used, and an External Quality Assurance (EQA) program was followed with Bio-Rad (Bio-Rad Laboratories, Inc., Hercules, California, United States). The total vitamin D levels were initially categorized as deficient (<20 ng/ml), insufficient (21-29 ng/ml), and sufficient (30-100 ng/ml) [14,15]. For the purposes of our analysis, we categorized the levels into two groups: adequate (≥30 ng/ml) and inadequate (<30 ng/ml).

Data analysis

Data were analyzed using IBM SPSS Statistics for Windows, Version 21.0 (Released 2012; IBM Corp., Armonk, New York, United States). Both continuous and categorical variables were presented using descriptive statistics of mean, standard deviation, and percentage. A chi-squared test, independent two-sample t-test, and one-way ANOVA were performed to assess the statistical relationship between vitamin D deficiency and the associated factors with a 95% confidence interval (CI).

## Results

Sociodemographic characteristics

All 371 recruited subfertile women participated in the study; thus, the respondent rate was 100%. The age range of the participants was 20-51 years, with a mean age of 33.7 years (SD=6.7), and nearly half of them (46.6%) were in the age group of 30-40 years. All the participants were Sri Lankan Tamil, and the majority (77.9%) were Hindus. Half of the participants (50.1%) had studied up to the Ordinary Level, 46% had a monthly family income of less than 23000 LKR, and nearly a three-quarter of the participants (72.5%) were unemployed (Table 1). 

**Table 1:** Distribution of sociodemographic factors among participants (N=371)

Sociodemographic factors	Category	Frequency	Percentage
Age (years)	20-30	132	35.6
	30-40	173	46.6
	>40	66	17.8
BMI (kg/m^-2^ )	<18.5	20	5.4
	18.5-24.9	144	38.8
	25-29.9	137	36.9
	30/>30	70	18.9
Ethnicity	Sri Lankan Tamil	371	100
Religion	Hinduism	289	77.9
	Christianism	82	22.1
Educational level	Up to Gr.10	49	13.2
	Up to Ordinary Level	186	50.1
	Up to Advanced Level	85	22.9
	Diploma/ Degree	51	13.7
Occupation	Part-time employed	18	4.9
	Full time employed	84	22.6
	Unemployed	269	72.5
Monthly income (in LKR)	<23000	178	48
	23000-46000	148	39.9
	46000-100000	44	11.9
	>100000	1	0.3

Subfertility-related factors

Nearly three-fourths of participants (72%) had primary subfertility, and almost half (52.6%) had one to three years of subfertile period. The subfertility period ranged from one to 22 years, with a mean duration of 4.70 years (SD=3.7). However, most of the participants (85.7%) were currently on subfertility treatment. Considering the etiology related to subfertility, more than half (56.3%) were affected by polycystic ovaries; 12.9% and 5.9% of the participants had fibroids and endometriosis, respectively. Further, 40.2% of the participants had irregular menstruation cycles (Table 2).

**Table 2:** Distribution of subfertility and vitamin D-related factors among participants (N=371)

Subfertility factors	Category	Frequency	Percentage
Type	Primary	267	72
	Secondary	104	28
Duration of subfertility (In years)	1-3	195	52.6
	3-10	134	36.1
	>10	42	11.3
Current treatment details	1^st^ visit	54	14.6
	Following	264	71.1
	Defaulted	53	14.3
Previous vitamin D deficiency	Yes	4	1.1
	No	10	2.7
	Not checked	357	96.2
Intake of (multi) vitamin supplements	Yes	304	81.9
	No	67	18.1
Exposure to sunlight (>30 minutes)	Yes	318	85.7
	No	53	14.3
Use of sun protection cosmetics	Yes	145	39.1
	No	226	60.9
Irregular period	Yes	149	40.2
	No	222	59.8
Polycystic ovary	Yes	209	56.3
	No	162	43.7
Endometriosis	Yes	22	5.9
	No	349	94.1
Fibroids	Yes	48	12.9
	No	323	87.1
Hyperprolactinemia	Yes	2	0.5
	No	369	99.5
History of head and neck trauma	Yes	15	4
	No	356	96
History of chemotherapy/radiotherapy	Yes	2	0.5
	No	369	99.5

Vitamin D level and related factors

Most of the participants (96.2%) never investigated their vitamin D level prior to the study, and about 81.9% of them took vitamin supplements regularly. Although most of them (85.7%) had continuous exposure to direct sunlight for more than 30 minutes, only 39.1% used sun protection cosmetics (Table 2). The study revealed that only 36.4% of subfertile women had adequate vitamin D levels, whereas 63.4% had inadequate levels of vitamin D on the Bio-Rad EQA.

Association of sociodemographic and subfertility factors with vitamin D levels

The statistical association between sociodemographic factors and the level of vitamin D was assessed using a chi-squared test. Results revealed that there was no significant association between the level of vitamin D and the assessed sociodemographic factors such as age, family income, education, and employment type. However, participants who had primary subfertility at less than 30 years of age and/or were doing part-time jobs with less than 23000 LKR as monthly income, had adequate levels of vitamin D compared with another category (Table 3).

**Table 3:** Influence of sociodemographic and sub-fertility factors on the level of Vitamin D (N=371)

Sociodemographic and subfertility factors	Level of vitamin D				Statistical tests				
	Inadequate (< 29 ng/ml)		Adequate (30-100 ng/ml)		X^2^	df			P
	Frequency	Percentage	Frequency	Percentage					
Age (years)									
< 30	75	56.8	57	43.2	4.766		2	0.092	
30-35	63	64.3	35	35.7					
>35	98	69.5	43	30.5					
Occupation									
Part-time	9	50	9	50	5.815		2	0.055	
Full time	62	73.8	22	26.2					
Unemployed	165	61.3	104	38.7					
Monthly income (in LKR)									
<23000	107	60.1	71	39.9	2.879		2	0.237	
23000-46000	96	64.9	52	35.1					
>46000	33	73.3	12	26.7					
Type of subfertility									
Primary	167	62.5	100	37.5	0.467		1	0.494	
Secondary	69	66.3	35	33.7					

The association between subfertility-related factors and the level of vitamin D was assessed using a t-test. The findings showed no statistical association between vitamin D level and the etiological factors of subfertility, such as PCOS, endometriosis, hyperprolactinemia, fibroids, irregular periods, and previous history of head and neck trauma. Despite the association, the mean value of vitamin D was high in the participants with irregular menstruation and fibroids. On the other hand, the mean of vitamin D was high in the participants who did not have a previous history of head and neck trauma, PCOS, endometriosis, and hyperprolactinemia. Moreover, individuals who regularly consumed fish, beef liver, cheese, yogurt, and mushrooms had higher mean levels of vitamin D. Specifically, the regular intake of fish (p=0.03) and beef liver (p=0.03) were significantly associated with higher vitamin D levels. Even though there was no statistically significant association, continuous (>30 minutes) exposure to sunlight and the use of sun-protective cosmetics resulted in high levels of vitamin D among the participants (Table 4).

**Table 4:** Distribution of Influencing factors on serum vitamin D level among the participants (N=371) (Based on t-test)

Factors		Mean	Standard Deviation	t	df	Sig (2-tailed)
Associate subfertility factors						
Irregular period	Yes	28.054	5.843	0.119	369	0.905
	No	27.981	5.818			
Previous history of head and neck trauma	Yes	27.8	4.7	-0.143	369	0.887
	No	28.019	5.868			
PCOS	Yes	27.955	5.788	-0.207	369	0.836
	No	28.082	5.879			
Endometriosis	Yes	28.005	4.922	-0.005	369	0.996
	No	28.011	5.878			
Hyperprolactinemia	Yes	26.8	0.283	-0.295	369	0.769
	No	28.017	5.835			
Fibroids	Yes	28.26	5.771	0.319	369	0.75
	No	27.973	5.835			
Intake of foods containing vitamin D						
Milk	Yes	27.857	5.715	-0.716	369	0.474
	No	28.316	6.035			
Fish	Yes	28.187	5.784	2.18	369	0.03
	No	25.572	5.888			
Egg	Yes	27.897	5.7	-0.793	369	0.429
	No	28.516	6.348			
Beef liver	Yes	29.311	5.301	2.177	369	0.03
	No	27.681	5.907			
Cheese	Yes	28.965	4.698	0.692	369	0.49
	No	27.965	5.87			
Yogurt	Yes	28.715	4.917	1.282	369	0.201
	No	27.798	6.058			
Mushrooms	Yes	29.844	5.329	0.957	369	0.339
	No	27.965	5.831			
Exposure to sunlight	Yes	28.217	5.804	1.678	369	0.094
	No	26.772	5.818			
Usage of sun protection cosmetics	Yes	28.292	5.502	0.745	369	0.457
	No	27.83	6.02			

Specifically, the ANOVA test demonstrated that BMI level was significantly associated with vitamin D levels (F3, 367= 7.041, p=0.000). Results showed that BMI was negatively associated with vitamin D levels (Table 5).

**Table 5:** The association between vitamin D and BMI (based on ANOVA)

BMI groups (kg/m^2^)		Mean	SD	ANOVA		
				Levene statistic	F	Sig
Underweight		30.065	4.738	0.547	7.041	0.00001
Normal weight		28.845	5.309			
Overweight		8.189	5.889			
Obese		25.357	6.23			

## Discussion

The current study focused on assessing the prevalence and associated risk factors of vitamin D deficiency among subfertile women. The mean age of the study participants was 33.7 years (SD=6.7) and ranged from 20 years to 51 years, which has a consistent pattern with a case-control study done in Sri Lanka (mean age 31.7) [6] and a comprehensive study from Italy (mean age 36.3, SD=4.4) [16]. 

In the present study, around half of the participants were studied up to an ordinary level, and 18.9% were obese. It contradicts a previous study that revealed 45.7% were qualified as advanced level, and only 10.5% were obese [11]. In our study, the mean BMI of the population was 26.3 kg/m^2^ (SD=6.1) and had a range of 13-72 kg/m^2^, which is higher than in the study by Pagliardini et al. (mean BMI 22.4, SD=3.6) [7]. Moreover, in their study, the analysis revealed that 69.5% of the participants had primary subfertility, and 7% of them had endometriosis. It is almost similar to our present study findings. However, in our population, more than half of them have PCOS, which is much higher than previous studies done in Italy [7] and Turkey [17].

The current study is, to the best of our knowledge, the first study conducted to assess vitamin D deficiency (<20 ng/ml) among the subfertile women in Northern Sri Lanka. The results indicated that 7.8% and 55.8% of subfertile women had vitamin D deficiency (<20 ng/ml) and insufficiency (<30 ng/ml), respectively, which demonstrates overall, 63.6% of subfertile women did not have an adequate level of vitamin D. These results are commensurate with limited published data on the level of vitamin D among subfertile women in Sri Lanka [12], contrary to a study in Pakistan that revealed that 68.4% of subfertile women had vitamin D deficiency [13]. Other studies have shown that vitamin D insufficiency is higher (77.4%) in Italy [7] and lower (27%) in Sweden [18]. The current study revealed no significant association between vitamin D levels and age and type of subfertility, which agrees with a previous study done in Italy [7]. Previous studies have shown that BMI and serum vitamin D levels were significant predictive parameters [7,19] Our study also confirms these findings (p < 0.001).

Vitamin D has been involved in the development of specific gynecological conditions affecting fertility such as endometriosis and PCOS [20]. Vitamin D plays one of the regulatory roles in the pathogenesis of uterine fibroids [17]. Previous studies have shown that endometriosis was positively associated with vitamin D levels. Another study revealed no significant difference between vitamin D levels and PCOS [17]. Despite these findings, our study has shown no significant association between vitamin D level and subfertility factors, mainly in PCOS, endometriosis, and fibroids.

Regarding vitamin D sources, the primary source is the cutaneous synthesis of sunlight exposure and dietary elements such as eggs, fish, fortified milk and yogurt, and vitamin supplements [18]. Our study found that vitamin D level was significantly influenced by the intake of fish (p=0.03) and beef liver (p=0.03). In contrast, a prospective cohort study revealed no difference in daily dietary vitamin intake on serum vitamin D levels [18]. Also, the same study found that vitamin D insufficiency was higher in women who reported staying “in the shade all the time” compared to those who reported staying “in the sun all the time”. However, our study found no significant differences between exposure to sunlight and serum vitamin D levels.

The current study revealed that vitamin D deficiency among subfertile women is considerably high. Even though it is a significant contributor to subfertility, the issue still needs to be addressed. (nearly 89% of the subfertile women never estimated their vitamin D level prior to this study). It was observed that overweight and obese subfertile women had a high chance of vitamin D deficiency stage compared with underweight and normal weight. In addition, the findings suggested that the regular intake of fish and eggs was statistically associated with high levels of vitamin D.

A 100% respondent rate shows the timely need for the study and the completeness of data. Laboratory analysis using a standard vacutainer technique by trained nurses and medical laboratory scientists reporting vitamin D levels using international standards was the additional strength of the study. Even though the study had considerable strengths, it had a few limitations, such as the study was conducted in the Northern region and most of the participants were Hindus, which might affect the generalizability of the finding to the country as the living area and religion influence the dietary patterns and habits of the women, which has a strong association with vitamin D levels. 

## Conclusions

Vitamin D deficiency can be caused by several factors and impact human reproductive function. The findings emphasized that the prevalence of Vitamin D is high in sub-fertile women in the northern part of Sri Lanka, especially in the overweight and obese category, and the issue is poorly addressed with an unmet crucial need. The study recommends vitamin D assessment at the initial point of subfertility workup, periodical monitoring of vitamin D levels in all sub-fertile women with particular attention towards the obese and overweight category, and a combination of pharmacological supplementation and dietary intervention to manage the deficiency might improve the fertility outcomes. Further, a multi-center prospective study will be more beneficial to study the effectiveness of vitamin D supplementation among sub-fertile women in Sri Lanka, which might be a potential solution to overcome the burden.

## Figures and Tables

**Figure 1:**
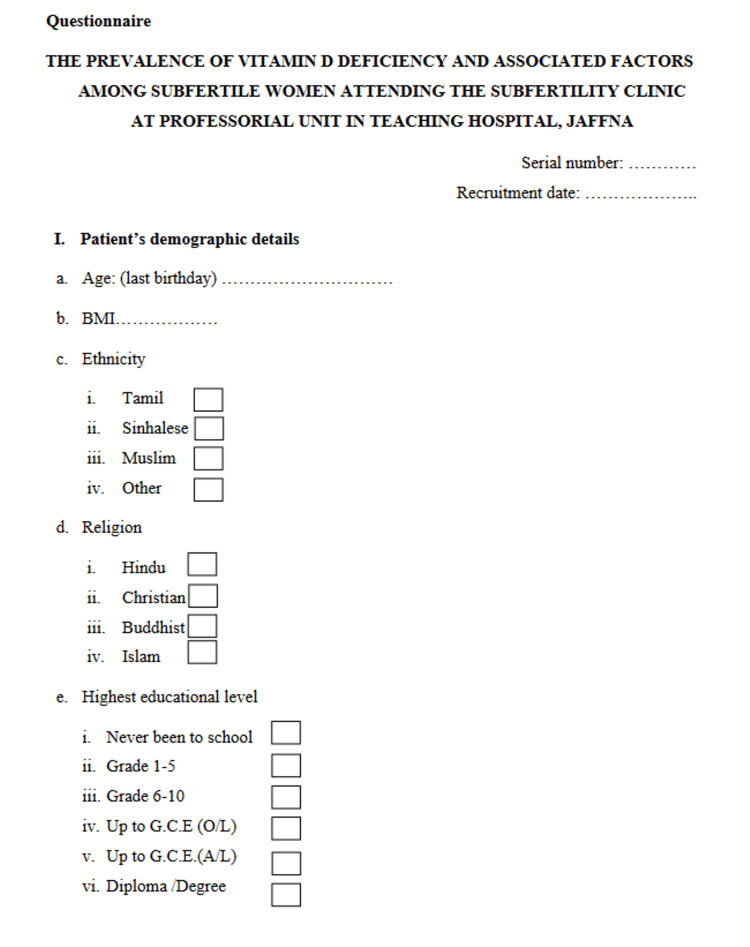
Questionnaire, page 1

**Figure 2:**
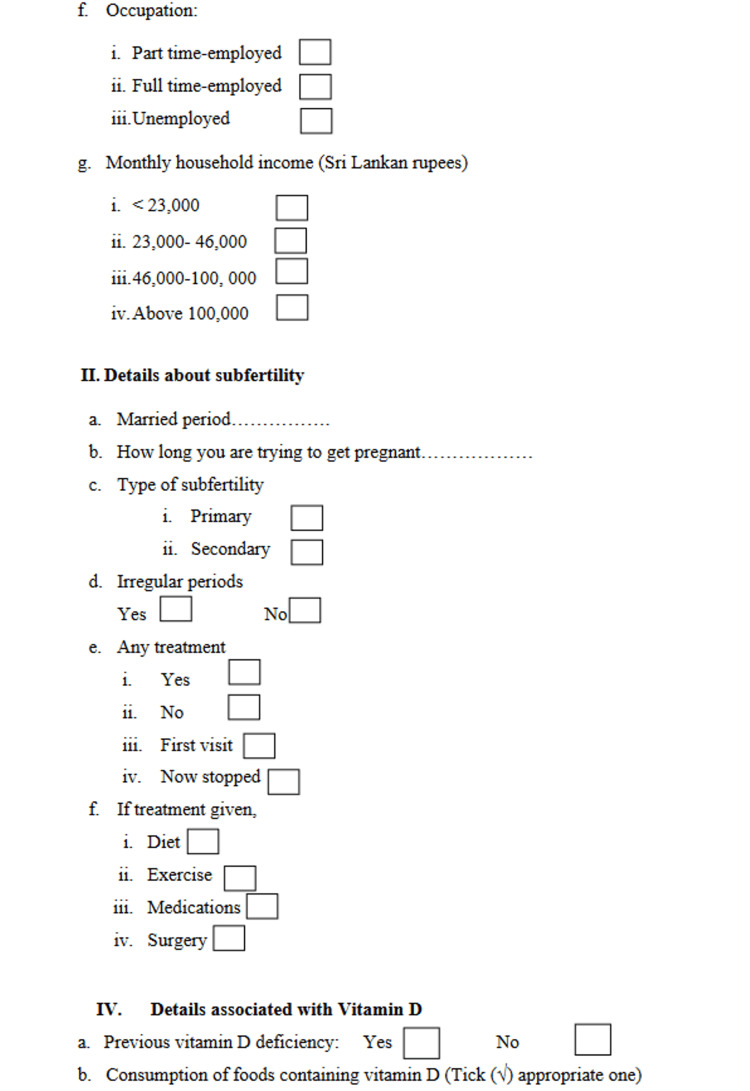
Questionnaire, page 2

**Figure 3:**
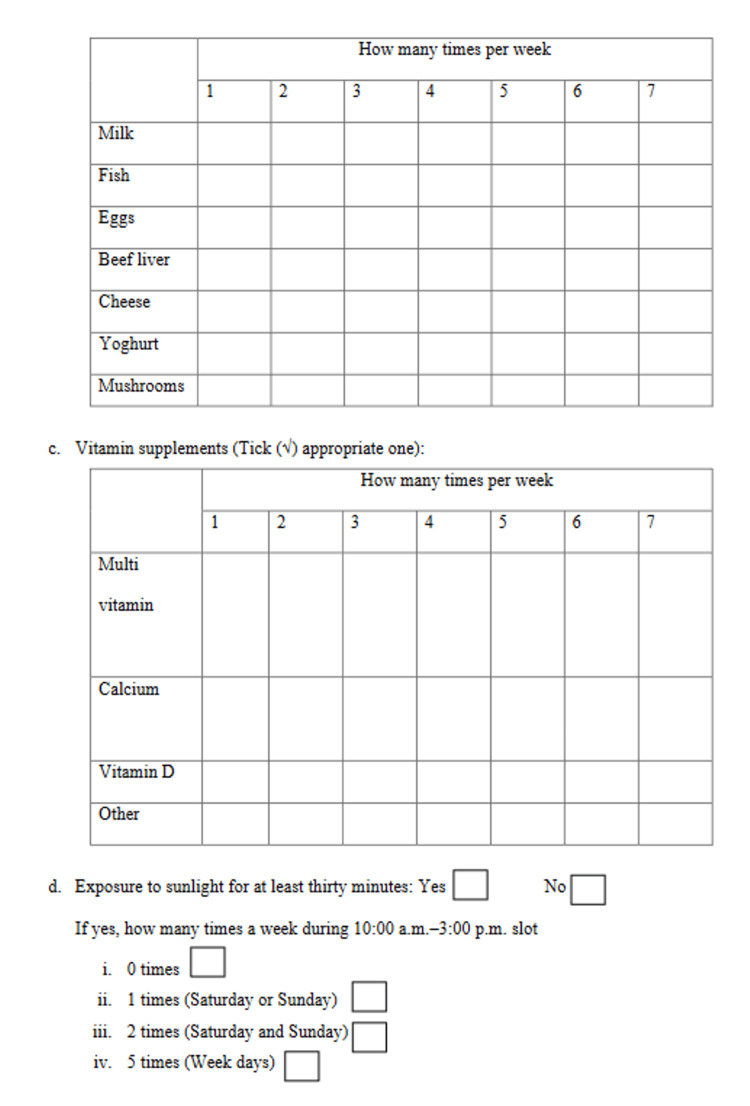
Questionnaire, page 3

**Figure 4:**
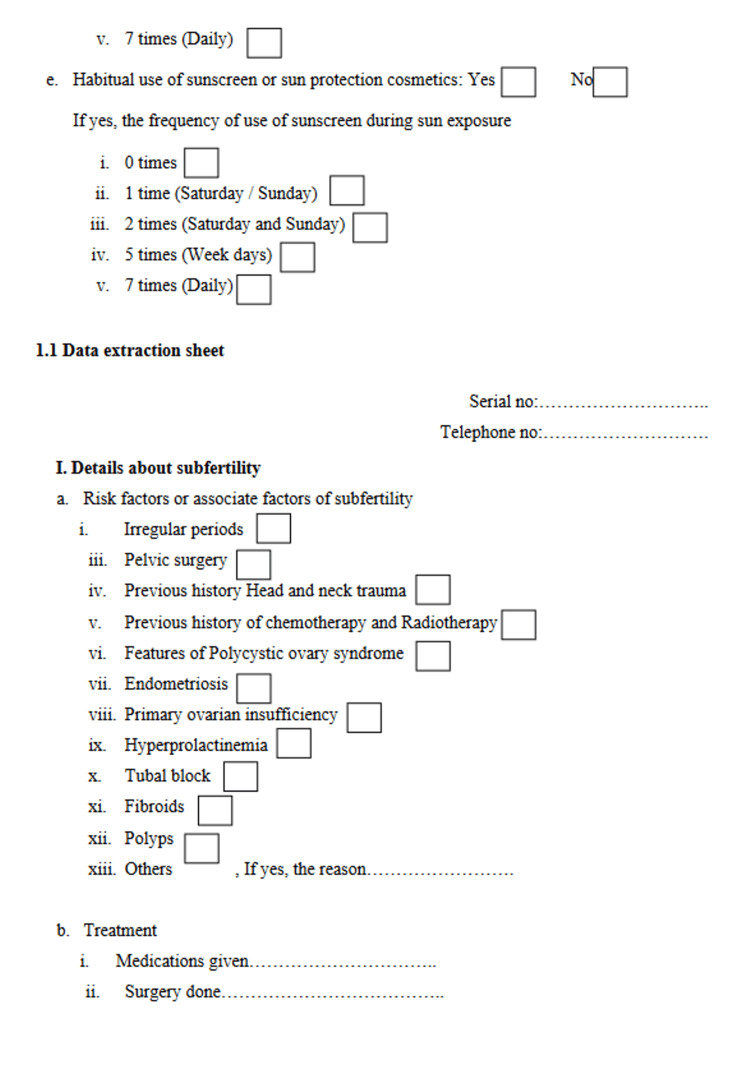
Questionnaire, page 4
